# Negative Incentives for Noninstitutional Births Are Associated With a Higher Rate of Facility-Based Births in the Eastern Visaya Region, Philippines

**DOI:** 10.9745/GHSP-D-20-00616

**Published:** 2021-09-30

**Authors:** Shogo Kanamori, Marcelyn D. Bonhaon, Minerva Peregrino Molon

**Affiliations:** a Department of Community and Global Health, Graduate School of Medicine, The University of Tokyo, Tokyo, Japan.; b Japan International Cooperation Agency, Department of Health, Project for Introducing Evidence-based Relapse Prevention Programs to Drug Dependence Treatment and Rehabilitation Centers in the Philippines, Manila, Philippines.; c Department of Health, Center for Development-Eastern Visayas, Palo, Leyte, Philippines.

## Abstract

In the Philippines, the “no home-birthing policy” implemented in 2008 has encouraged local governments to endorse ordinances that prohibit noninstitutional deliveries (NIDs). The Philippines’ social insurance scheme has also enabled them to provide incentive payments to women who deliver at birthing facilities (BFs). This study evaluated whether these positive and/or negative incentives were associated with an increased facility-based delivery (FBD) rate and examined challenges in implementing those incentive measures in the Eastern Visaya Region of the Philippines. Cross-sectional data were collected regarding delivery locations during 2017, and barangay-level data were collected regarding positive and negative incentives and the characteristics of 4,371 barangays in the region. Multivariate logistic regression analysis, using “ordinance with nonmonetary sanction” as the reference category, indicated that no ordinance to prohibit NIDs was significantly associated with a lower FBD rate (odds ratio [OR]: 0.90, *P*=.013), and ordinance with low cash penalty (OR: 1.37, *P*<.001) and high cash penalty (OR: 2.52, *P*<.001) had higher FBD rates. Positive incentives were not associated with FBD rates (OR: 1.02, *P*=.563). The FBD rate was also significantly associated with urban barangay status (OR: 1.45, *P*<.001), availability of a vehicle (OR: 3.19, *P*<.001), availability of public transportation to a government BF (OR: 1.25, *P*<.001), and distance to the most accessible government BF (OR: 0.89, *P*<.001). Substantial proportions of barangays had no vehicles available for transportation (34.1%) or no available vehicles and no public transportation to a government BF (5.5%). Although negative incentives for NIDs could motivate pregnant women to seek FBD, local governments in the Philippines should be aware of barriers that limit access to government BFs in underserved barangays before introducing those negative incentives. Above all, barangay-level investments in making a vehicle for transporting pregnant women might help address these barriers and increase the FBD rate.

## Abstract (precis)

Penalties imposed for noninstitutional deliveries by local government policies could motivate pregnant women to deliver at birthing facilities; however, local governments should address barriers to accessing a birthing facility in underserved areas before prohibiting noninstitutional deliveries.

## Teaser

**Key Findings:**

- Negative incentives to prohibit noninstitutional deliveries were significantly associated with higher facility-based delivery rates.
- No significant association was found between positive incentives and the facility-based delivery rate.

**Key Implications:**

- Along with introducing penalties for noninstitutional deliveries, investing limited resources to make a vehicle available at the barangay level could effectively address these barriers and increase the facility-based delivery rate.

## INTRODUCTION

Access to quality maternal care throughout pregnancy is the key to reduce preventable mortality and morbidity among pregnant women.^1^ In particular, facility-based deliveries (FBDs) are considered a crucial contributor to improving the maternal mortality ratio in low- and middle-income countries (LMICs).^2^^–^^4^ In the Philippines, the Department of Health (DOH) issued the Maternal, Newborn and Child Health and Nutrition (MNCHN) Strategy in 2008 to reduce maternal mortality by primarily promoting FBDs.^5^ The strategy was further updated by the MNCHN Manual of Operations, which was released in 2011 and contained a key statement on the prohibition of deliveries assisted by traditional birth attendants (TBAs).^6^ It particularly highlighted that deliveries assisted by TBAs contributed to the 3 delays, namely delays in identification of complications, referral, and management of complications, and led to maternal and neonatal deaths.^6^ These documents have been interpreted as a “no home-birthing policy” and have generated controversy regarding their appropriateness, especially in underserved small villages that are known as barangays.^7^^,^^8^ The documents resulted in several local governments endorsing ordinances to prohibit home-based deliveries assisted by TBAs and often to penalize TBAs and/or pregnant women who participate in noninstitutional deliveries (NIDs).^7^ The penalties indicated in the ordinances were either cash payments or nonmonetary sanctions such as a reprimand by authorities. Some ordinances simply prohibit home-based deliveries without specifying penalties.

The Philippines has a social insurance scheme (PhilHealth) with a Maternal Care Package that covers essential health services during the prenatal period and throughout all stages of labor, normal delivery, and the immediate postpartum period. Cases that involve a normal delivery are reimbursed at rates of 8,000 Philippine pesos (PHP) for accredited primary care facilities and 6,500 PHP for hospitals.^9^ Local governments with jurisdiction over those facilities may decide to direct a portion of the reimbursed amount to the women as a cash incentive to promote FBDs.

Individual- and community-level factors that affect FBDs have been examined in LMICs, and these factors are related to the local sociodemographic, socioeconomic, and cultural situation.^10^^–^^14^ A study by Bohren et al.^14^ indicated that key barriers to FBD include traditional and familial influences, distance to the facility, cost of delivery, low perceived quality of care, and fear of discrimination during FBD.^14^ In the Philippines, a study based on the 2013 National Demographic Health Survey^15^ revealed that the FBD rate might be influenced by the child’s birth order, the mother’s religion, and the subjective distance to the closest health care facility.^16^ Another study revealed that a woman‘s choice of FBD might be influenced by the involvement of the husband and other people in the decision regarding delivery location.^17^

Financial incentives and penalties have also been used to promote maternal care service utilization and FBDs in LMICs. A systematic review by Murray et al.^18^ identified various studies that examined how demand-side financing or positive incentives influenced women’s decision to seek the services of a skilled birth attendant. A few studies also examined the impacts of the penalties or negative incentives that were applied to TBA-assisted deliveries.^19^^,^^20^ A qualitative study in the Philippines also examined mothers’ and service providers’ views regarding the effects of financial incentives and penalties on the FBD rate.^21^ Despite these studies of antecedents that focused on the positive and negative incentives, to the best of our knowledge, little has been studied about the effects of those incentives on the FBD rate in the Philippine context. In addition, few studies have quantitatively examined the association between the negative incentive for NID and the increase of the FBD rate in LMICs.

Despite studies of positive and negative incentives, little has been studied about the effects of such incentives on the facility-based delivery rate in the Philippines.

This study evaluated the association between local governments’ policies regarding positive and negative incentives to promote FBD and pregnant women’s decisions to seek FBDs and NIDs in the Eastern Visaya Region of the Philippines. The study also examined challenges in implementing those incentives and policy measures that might be useful for increasing the FBD rate in the region.

## METHODS

### Target Areas and Facilities

The Eastern Visaya Region is 1 of the 17 regions in the Philippines, with a population of 4,440,150 people. The region is composed of 6 provinces that are subdivided into 143 cities/municipalities and then into 4,390 barangays.^22^ The region’s total land area is 23,234.8 km^2^, which accounts for 7.7% of the national land area.^23^ The per capita gross domestic product (GDP) in the Eastern Visaya Region was 73,995 PHP or US$1,404 in 2018, while the national average per capita GDP was 163,474 PHP or US$3,102.^24^

Institutional delivery takes place at birthing facilities (BFs), which are categorized as hospitals, infirmaries (quasi-hospitals that do not satisfy the DOH’s hospital standards), or birthing homes (primary care facilities that satisfy the DOH’s standard for BFs). As of December 31, 2016, the Eastern Visaya Region included 46 hospitals (23 government and 23 private hospitals), 38 infirmaries (28 government and 10 private centers), and 127 birthing homes (104 government and 23 private centers).^25^^–^^27^ Each barangay has a barangay health station (BHS), which is the most peripheral health facility that provides primary health care services, including antenatal care. Functional BHSs are staffed with a full-time or part-time rural health midwife (RHM) or a nurse, although some BHSs are considered nonfunctional because adequate staff are not available. All the women in the region were exempted from paying for delivery services at government facilities during the study period because of the devastation caused by Typhoon Haiyan in 2013, indicating that the user fee payment is less likely to be a barrier to FBD for pregnant women in this study.^28^

### Data Collection

Cross-sectional data of deliveries in 2017 were collected between February and May 2018 from the 4,390 barangays in Eastern Visaya Region. The 1,568 temporary staff who had been hired and assigned at the barangay level under the DOH’s deployment program (Nurse Deployment Program: 1,465 individuals, Universal Health Coverage Implementor Deployment Program: 103 individuals) were trained to serve as data collectors.^29^ The collected data were compiled at the municipality/city level and submitted to the DOH research team in Manila; data of 19 barangays were not submitted. Thus, the present study included data of 4,371 barangays, which were entered into a database for analysis.

Cross-sectional data of deliveries in 2017 were collected between February and May 2018 from the 4,390 barangays in Eastern Visaya Region.

A questionnaire form was used to collect 3 types of data at the barangay level.

First, delivery-related statistics were gathered. FBDs and NIDs that involved each barangay’s residents were identified between January 1 and December 31, 2017. In principle, the BHS-level prenatal registers are filled out by RHMs or nurses in charge of the barangays. The prenatal register is updated each time barangay health workers (BHWs) identify a new pregnant client, when BHWs conduct house-to-house visits for pregnancy tracking in their barangays, when a registered pregnant woman makes a prenatal visit or gives birth, or when a nonregistered woman gives birth at a facility located within the barangay. Information of NIDs and deliveries that occur at private facilities and government facilities in other municipalities/cities is supposed to be relayed to the RHMs or nurses in charge and retrospectively reflected on the BHS-level prenatal registers. However, in reality, the BHS-level prenatal registers have 3 issues: (1) they often fail to record information regarding deliveries by pregnant residents that occur in other municipalities/cities, (2) they occasionally do not include pregnant residents who did not seek antenatal care at the BHS, and (3) they include nonresidents who delivered at a facility located within the barangay. Therefore, to meet the purposes of the study, a register that only considered deliveries by residents in their own barangay was introduced to guide the barangay-level calculations of FBDs and NIDs.^30^ Information gaps regarding pregnant residents who did not appear in the prenatal registers were filled based on the recall of the RHMs or nurses in charge of the barangays, as well as in consultation with BHWs who were familiar with the household statuses of barangay residents from their periodical house visits.

Second, missed delivery cases were accounted for. To estimate the chance of missed delivery cases in the barangays, the RHMs or nurses in charge were asked to answer the following question: “Have you ever come across a woman among the barangay residents whose pregnancy status you were not aware of during the prenatal period, but became aware of the pregnancy history only when a newborn child was brought to you for immunization or other services?” If the answer was “yes,” they were additionally asked to give the number of such cases they observed in 2017.

Third, barangay characteristics were recorded. The RHMs or nurses in charge of the barangays were consulted to answer the following questions: (1) “Did pregnant women in this barangay receive any incentive payments for FBDs during 2017?” and (2) “Was there a local ordinance or policy supported by a written official order to prohibit NIDs that was effective during 2017?” In connection with the second question, the type and cash amount of the penalties were also asked. Additional information was collected regarding whether the barangay was classified as urban or rural; whether an RHM or a nurse was deployed at the BHS (full-time/part-time or none); what was the most accessible government BF; whether a vehicle was available for transporting pregnant women; and whether a government BF was accessible via public transportation (or walking). A vehicle was considered available if (1) the barangay owned a functional ambulance/vehicle, (2) an ambulance/vehicle owned by the municipality/city/province would pick up pregnant women within the barangay, (3) the barangay had an official memorandum of agreement with private health care facilities to use their ambulance/vehicle, or (4) the barangay had an official memorandum of agreement with owners of personal vehicles or tricycles. The distance from the barangay centroid to the most accessible government BF was also calculated for each barangay.

### Data Analysis

The barangay-level dataset was reorganized to generate a new individual-level dataset of deliveries during the study period that were coded as FBDs (a value of 1) or NID (a value of 0); all the delivery cases in the new dataset inherited the variables of the barangay characteristics. A multivariate logistic regression analysis was performed with the binary status of FBD as the outcome variable and the barangay’s characteristics as the explanatory variables. The barangay’s characteristics used in the logistic regression model included (1) providing incentive payments for FBDs, (2) local ordinance to prohibit NIDs, (3) urban or rural barangay status, (4) an RHM or nurse deployed (full-time/part-time or none), (5) vehicle availability, (6) public transportation availability, and (7) distance to the most accessible government BF. Among these variables, the local ordinance to prohibit NIDs was treated as a categorical variable with the following categories that were dummy coded: (1) yes, nonmonetary sanction or penalty not specified, (2) yes, with low cash penalty, (3) yes, with high cash penalty, and (4) no. All the other variables were treated as binary variables, except the distance to the most accessible government BF, which was treated as a continuous variable. Odds ratios (ORs) of each variable were calculated with 95% confidence intervals (CIs) and *P*-values. The variance inflation factors were also calculated to detect the presence of multicollinearity in the model. Additionally, we analyzed the vehicle availability at the barangay level by its ownership.

### Ethical Considerations

The study protocol was exempted from review by the Research Ethics Review Committee of Eastern Visayas Health Research and Development Consortium, as it was considered low-risk research that did not involve direct contact with human subjects. Client-level data were de-identified during the data aggregation process. No direct contact was made with pregnant women throughout the study period.

## RESULTS

During the study period, we identified 74,414 deliveries in the 4,371 barangays of the Eastern Visaya Region. The deliveries included 65,842 FBDs, 6,016 NIDs, and 2,556 deliveries at unknown places. When we only considered deliveries in known locations, the regional FBD rate was 91.6% (Table 1). In addition, during the immunization service provided in the 4,371 barangays in the study period, 4,228 delivery cases that had not previously been reported by the BHWs were identified.

**TABLE 1.** Distribution of Deliveries That Occurred During 2017 Among Pregnant Women Residing in 4,371 Barangays in the Eastern Visaya Region, Philippines

Province	Total Deliveries	FBD Cases	NID Cases	Delivery Place Unknown	Delivery Place Known	
					Total	FBD Rate, %
Biliran	3,458	3,365	80	13	3,445	97.7
Eastern Samar	7,757	7,014	537	206	7,551	92.9
Leyte	31,958	30,196	760	1,002	30,956	97.5
Northern Samar	10,488	8,002	2,037	449	10,039	79.7
Samar	14,585	11,295	2,558	732	13,853	81.5
Southern Leyte	6,168	5,970	44	154	6,014	99.3
Total	74,414	65,842	6,016	2,556	71,858	91.6

Abbreviations: FBD, facility-based delivery; NID, noninstitutional delivery.

Table 2 shows the characteristics of the 4,371 barangays. The results indicated that 43.2% of the barangays provided incentive payments for FBDs and 77.6% had implemented a local ordinance or policy supported by a written official order to prohibit NIDs. The vast majority of barangays were rural (97.3%) and 10.4% of barangays had nonfunctioning BHSs (i.e., no staff to provide routine maternal care). A vehicle was considered available for transporting pregnant women in 65.9% of the barangays, and most barangays (91.3%) had available public transportation to government BFs. However, 5.5% of the barangays did not have an available vehicle or public transportation. The median distance from the barangay centers to the most accessible BF was 4.06 km (range: 0–39.4 km, interquartile range: 5.54 km). Among barangays that provided incentive payments for FBD, the mean value was 1,239 PHP (range: 10–8,000 PHP, standard deviation: 541 PHP). Concerning the penalties for the first-time offense of NIDs, 20.6% either indicated nonmonetary sanction or did not specify any penalty in the orders, 28.5% indicated cash penalty amounts ranging between 200 and 1,000 PHP, and 28.5% between 1,200 and 10,000 PHP. The orders were supported by city, municipality, and/or barangay-level ordinances.

**TABLE 2.** Characteristics of the 4,371 Barangays That Were Included in the Study Looking at Incentives and Institutional Deliveries in Eastern Visaya Region, Philippines, 2017

Questionnaire Items	No.	%
Pregnant women receiving any incentive payment for FBDs		
Yes	1,848	43.2
No	2,433	56.8
Missing	90	—
Existence of a local ordinance or policy supported by a written official order to prohibit NID		
Yes, nonmonetary sanction or no specific penalty	881	20.6
Yes, with low cash penalty (200–1,000 PHP)	1,218	28.5
Yes, with high cash penalty (1,200–10,000 PHP)	1,221	28.5
No	957	22.4
Missing	94	—
Barangay classification		
Urban	119	2.7
Rural	4,252	97.3
Assignment of a full-time or part-time RHM or nurse for routine maternal care services in the barangay		
Yes	3,741	89.6
No	436	10.4
Missing	195	—
A functional vehicle available to transport pregnant women		
Yes	2,812	65.9
No	1,453	34.1
Missing	106	—
Public means of transport to a government BF available		
Yes	3,936	91.3
No	376	8.7
Missing	59	—

Abbreviations: BF, birthing facility; FBDs, facility-based deliveries; NID, noninstitutional delivery; PHP, Philippine pesos; RHM, rural health midwife.

Multivariate logistic regression analysis revealed that FBD was significantly associated with barangays prohibiting NIDs. When using “yes, nonmonetary sanction or no specific penalty” as the reference category, FBD was negatively associated with barangays without an ordinance to prohibit NIDs (OR: 0.90, 95% CI: 0.83–0.98), *P*=.013) and positively associated with low cash penalty (200–1,000 PHP) (OR: 1.37, 95% CI: 1.26–1.49, *P*<.001) and high cash penalty (1,200–10,000 PHP) (OD: 2.52, 95% CI: 2.29–2.78, *P*<.001). Moreover, FBD was not associated with barangays providing an incentive payment for FBDs (OR: 1.02, 95% CI: 0.96–1.09, *P*=.563) (Table 3). In addition, FBD was associated with urban barangay status (OR: 1.45, 95% CI: 1.27–1.67, *P*<.001), availability of a vehicle for transporting pregnant women (OR: 3.19, 95% CI: 3.00–3.39, *P*<.001), availability of public transportation to government BFs (OR: 1.25, 95% CI: 1.13–1.39, *P*<.001), and distance (km) to the most accessible government BF (OR: 0.89, 95% CI: 0.89–0.90, *P*<.001) (Table 3). Running a regression using the interaction term between the ordinance and distance, we found that the directions of the associations between FBD and all the other variables, including the ordinance and the distance, unchanged from the regression result without the interaction term. The variance inflation factors calculated for each variable ranged between 1.02 and 1.84 (mean: 1.30), indicating a negligible level of multicollinearity.

**TABLE 3.** Logistic Regression Analysis to Measure the Associations Between Facility-Based Delivery and the Characteristics of 4,371 Barangays in the Eastern Visaya Region, Philippines

	Odds Ratio	95% CI	*P* value
Incentive payment provided for FBD	1.02	0.96, 1.09	.563
Ordinance to Prohibit NID			
No	0.90	0.83, 0.98	.013
Yes, nonmonetary sanction or no specific penalty	(Reference)		
Yes, with low cash penalty (200–1,000 PHP)	1.37	1.26, 1.49	<.001
Yes, with high cash penalty (1,200–10,000 PHP)	2.52	2.29, 2.78	<.001
Urban barangay	1.45	1.27, 1.67	<.001
RHM/nurse available (full-time or part-time)	1.07	0.95, 1.20	.298
Vehicle available	3.19	3.00, 3.39	<.001
Public transportation to government BF	1.25	1.13, 1.39	<.001
Distance to the most accessible government BF, km	0.89	0.89, 0.90	<.001

Abbreviations: BF, birthing facility; CI, confidence interval; FBD, facility-based delivery; NID, noninstitutional delivery; PHP, Philippine pesos; RHM, rural health midwife.

Multivariate logistic regression analysis revealed that FBD was significantly associated with barangays prohibiting NIDs.

Table 4 shows details of the vehicle availability at the barangay level by ownership. Among the 4,371 barangays, 7.0% had a functional ambulance/vehicle, 64.2% had access to an ambulance/vehicle owned by the municipality/city/province, 3.0% had an agreement with private facilities to use an ambulance/vehicle, and 2.0% had an agreement with personal vehicle owners.

**TABLE 4.** Details of Vehicle Availability at the Barangay Level by Ownership, in the Eastern Visaya Region, Philippines (N=4,371)

	No.	%
Barangay having a functional ambulance/vehicle		
Yes	302	7.0
No	3,998	93.0
Missing	71	—
An ambulance/vehicle owned by the municipality/city/province available		
Yes	2,757	64.2
No	1,538	35.8
Missing	76	—
Barangay having an agreement with private facilities to use an ambulance/vehicle		
Yes	130	3.0
No	4,135	97.0
Missing	106	—
Barangay having an agreement with personal vehicle owners		
Yes	84	2.0
No	4,190	98.0
Missing	97	—

## DISCUSSION

Our study found that ordinances to prohibit NIDs, even with nonmonetary sanctions such as reprimand or without specific penalties, were significantly associated with higher FBD rates. It also revealed that the penalty amount indicated in the ordinances was positively associated with the FBD rate. However, the study results indicated no significant association between positive incentives and the FBD rate. In addition, the FBD rate was higher in barangays that had urban status, vehicles available to transport pregnant women, public transportation available to government BFs, and were located a shorter distance to the most accessible government BF.

Ordinances to prohibit NIDs, even with nonmonetary sanctions such as reprimand or without specific penalties, were significantly associated with higher FBD rates.

Several studies have examined whether positive incentives influence FBDs in LMICs,^18^ although there is less information regarding whether cash payments contingent on FBD influence the birth location. This study focused on such an unstudied area and revealed that positive incentives were not significantly associated with the FBD rate, which may suggest that the contingent payment of cash incentives is not effective at motivating women to use BFs for FBDs. However, the cross-sectional nature of this study highlights the importance of caution when interpreting the causality of this relationship. For example, local governments may be aware of low FBD rates in their areas, which could motivate them to provide incentive payments for FBD in an attempt to improve their FBD rate. Thus, a longitudinal study is needed to clarify the effects of positive incentives on the FBD rates in the Philippines.

Several studies have also examined the effects of penalties on FBDs,^19^^–^^21^ although we are not aware of any studies that have quantitively identified an association between negative incentives and FBDs. Thus, our study serves to fill the knowledge gap regarding this relationship in the Philippines, which in 2008 implemented a no home-birthing policy^5^ that had been locally endorsed in 3,320 barangays (>75% of the Eastern Visaya Region) by 2017. Apparently, the association between negative incentives and FBDs could be explained by pregnant women’s behavioral patterns to avoid penalties. However, a qualitative study conducted in the Eastern Visaya Region of the Philippines also indicated stigma, shame, and fear borne in the culture at the barangay level out of the ordinances prohibiting NIDs as significant contributors to driving the women toward FBDs.^21^ Despite the novelty of our study findings, some areas still need to be further researched. First, our study dataset did not provide information about how many women among those who engaged in NIDs were aware of the existence of ordinances and the penalty amounts. The FBD rate could have been more significantly associated with the ordinances and the penalty amounts among women who were aware of their existence and the amounts. Second, we were unable to provide information about how many NID cases were actually ordered to pay a fine. It is particularly interesting to understand to what extent the existence of ordinances is effective as a deterrent to NIDs even without levying a fine. In addition, in this region, negative incentives were associated with a higher FBD rate, while positive incentives did not influence FBDs. There is no straightforward explanation to support this finding; however, this result might be justified by the prospect theory proposed by Kahneman and Tversky,^31^ which states that losses cause a greater emotional impact on an individual than does an equivalent amount of gain.

The study results imply that the no home-birthing policy helped lower the NID rates and increased the FBD rates. Nevertheless, concerns remain regarding the appropriateness of levying penalties for NIDs. Especially for indigent individuals, the cash penalty that ranges up to 10,000 PHP could be catastrophic. The Gabriela Women’s Party, a Filipino organization that advocates for women’s issues, also expressed concern that the no home-birthing policy may even result in an increase in the incidence of maternal and infant deaths by presenting a case of a pregnant woman in labor who tried to walk and cross a river and died before reaching the nearest BF.^7^ The present study also identified several barriers to FBDs in underserved barangays, which should be considered and addressed before prohibiting NIDs and financially penalizing women who seek them.

The results imply that the no home-birthing policy helped lower NID rates and increased FBD rates, but penalties for NIDs could be inappropriate.

One of the barriers to increasing FBDs was the accessibility of the BFs, as 34.1% of the barangays did not have a vehicle available to transport pregnant women and 5.5% of the barangays did not have an available vehicle or public transportation that would allow women to access a government BF. Furthermore, the distance from the center of some barangays to the most accessible BF was >30 km in some instances. Moreover, logistic regression analysis revealed that a higher FBD rate was significantly associated with vehicle availability, public transportation availability, and a shorter distance to a BF, with vehicle availability being a very strong determinant (OR: 3.19). These results have 2 policy implications. First, measures that penalize NIDs should be implemented with caution, as they may be unethical in barangays where the lack of a vehicle or public transportation makes the BF difficult to access. Second, with limited resources, investing in a vehicle to transport women to a BF might be an appropriate measure to increase the FBD rate, given that it is likely simpler and more realistic than improving public transportation availability or shortening the distance to a BF. Despite the cross-sectional nature of the study, our interpretation of the causal relationship between the FBD rate and the vehicle availability could be supported by a systematic review study conducted in LMICs, in which the poor availability of transportation was identified as a crucial factor in women’s decision to deliver at a facility.^14^ To increase the FBD, it would be ideal for all the barangays to have a functional vehicle; however, with the limited barangay budget composed of the Internal Revenue Allotment from the national government and nominal revenues at the barangay level,^32^ it may not be realistic. In fact, we found in our study that only 7.0% of the barangays owned a functional vehicle. An alternative and realistic measure could be to provide a vehicle at the municipality, city, and provincial levels as evidenced in our study results that a majority of the barangays with a vehicle available to transport pregnant women made such an arrangement.

Interestingly, the FBD rate was not significantly associated with the local availability of an RHM or nurse, even though 10.4% of the barangays did not have staff to support the BHS. In this context, pregnant women in the Philippines are required to visit an RHM or nurse for antenatal care at least 4 times during the prenatal period.^33^ However, women in barangays with nonfunctional BHSs would need to visit another barangay for their antenatal care, which is likely associated with an economic burden and a decreased likelihood of compliance with the antenatal care requirement. Further studies are needed to understand how understaffing at the barangay level influences outcome indicators, such as antenatal care coverage, and the results might be of interest to policymakers.

### Limitations

This study has several limitations. First, the study’s cross-sectional nature precludes a conclusion regarding the causality of the relationships between the FBD rate and the other variables. Second, the study primarily identified deliveries based on prenatal registers that were maintained at the BHSs, and missing data or missed deliveries are possible, especially in barangays with poor pregnancy tracking. The number of missing data represented by “delivery place unknown” in our study was 2,556, which accounted for 3.4% of the total deliveries. In addition, during the immunization service in 2017, health workers found 4,228 missed deliveries that occurred in 2016 or early 2017. Assuming that the same level is applied to the study period and that a majority of the missed cases were captured during the immunization visits, the missed deliveries could account for around 5.7% of the total deliveries. We cannot exactly identify reasons for the missing data and the missed deliveries; however, they may presumably include pregnant women using private facilities, BHWs’ insufficient performance or deployment in conducting the pregnancy tracking, and urbanized society with attenuated neighbor relations that make it difficult for the BHWs to identify residents’ pregnancy and delivery statuses.

## CONCLUSION

The findings from this study suggest that negative incentives for NIDs might help increase the FBD rate at the barangay level, although positive incentives were not associated with an increase in the FBD rate. Nevertheless, the appropriateness of the no home-birthing policy must be carefully assessed, as we identified several barriers that may reduce access to BFs and limit the FBD rate in underserved barangays. These barriers include the nonavailability of a vehicle or public transportation to help pregnant women visit BFs, as well as long distances to the nearest BF. Therefore, local governments in the Philippines should be aware of these barriers when planning or implementing penalties for NIDs. Above all, with limited resources, investing in a vehicle to transport pregnant women to BFs might be an effective and realistic measure to address these barriers and increase the FBD rate.

## Notes

**Peer Reviewed**

**First published online:** July 26, 2021.
